# Hypertension and cardiac damage in pheochromocytoma and paraganglioma patients: a large-scale single-center cohort study

**DOI:** 10.1186/s12872-024-03936-6

**Published:** 2024-06-26

**Authors:** Yang Yu, Chuyun Chen, Lei Meng, Wencong Han, Yan Zhang, Zheng Zhang, Ying Yang

**Affiliations:** 1 Department of Cardiology, Peking University First Hospital, Beijing, China; 2 Echocardiography Core Lab, Institute of Cardiovascular Disease, Peking University First Hospital, Beijing, China; 3 Department of Urology, Peking University First Hospital, Beijing, China; 4 Institute of Cardiovascular Disease, Peking University First Hospital, Beijing, China; 5 Institute of Urology, Peking University, Beijing, China; 6 National Urological Cancer Center, Peking University First Hospital, Beijing, China

**Keywords:** Pheochromocytoma, Paragangliomas, Hypertension, Left ventricular mass index, Left ventricular hypertrophy

## Abstract

**Background:**

Hypertension (HT) is one of the most common manifestations in patients with catecholamine-secreting neuroendocrine tumors. Although the cardiovascular manifestations of these tumors have been described, there have been no large-scale investigations of the profile of HT and changes in cardiac structure and function that occur in patients with pheochromocytomas and paragangliomas (PPGL).

**Materials and methods:**

In this study, we investigated the prevalence of HT and left ventricular remodeling (LVR) in a cohort of 598 patients who underwent surgery for PPGL at our center between January 2001 and April 2022. Information on demographics, reason for hospitalization, medical history, biochemical parameters, findings on echocardiography, and tumor characteristics were recorded. The LVR index was compared according to whether or not there was a history of HT.

**Results:**

The average age was 47.07 ± 15.07 years, and 277 (46.32%) of the patients were male. A history of HT was found in 423 (70.74%) of the 598 patients. Paraganglioma was significantly more common in the group with HT (26.00% vs. 17.71%, *P* = 0.030) and significantly less likely to be found incidentally during a health check-up in this group (22.93% vs. 59.43%, *P* < 0.001). Among 365 patients with complete echocardiography data, left ventricular mass index (86.58 ± 26.70 vs. 75.80 ± 17.26, *P* < 0.001) and relative wall thickness (0.43 ± 0. 08 vs. 0.41 ± 0.06, *P* = 0.012) were significantly higher in patients with PPGL and a history of HT. The proportions with left ventricular hypertrophy (LVH) (19.40% vs. 8.25%, *P* = 0.011) and LVR (53.73% vs. 39.18%, *P* = 0.014) were also higher when there was a history of HT. After adjusting for age, gender, body mass index, alcohol consumption, smoking status, diabetes, stroke, creatinine level, tumor location, and tumor size, a history of HT was significantly correlated with LVH (odds ratio 2.71, 95% confidence interval 1.18–6.19; *P* = 0.018) and LVR (odds ratio 1.83, 95% confidence interval 1.11–3.03; *P* = 0.018).

**Conclusion:**

HT is common in patients with PPGL (70.74% in this cohort). PPGL without a history of HT is more likely to be found incidentally (59.43% in our cohort). HT is associated with LVR in PPGL patients with complete echocardiography data. These patients should be observed carefully for cardiac damage, especially those with a history of HT.

**Supplementary Information:**

The online version contains supplementary material available at 10.1186/s12872-024-03936-6.

## Background

Pheochromocytomas and paragangliomas (PPGL) are catecholamine-producing neuroendocrine tumors for which surgery is still the only curative therapy. Hypertrophic cardiomyopathy in PPGL patients could have serious consequences and be fatal in some cases [1, 2]. In most cases, cardiac damage in PPGL involves long-term changes in the structure and function of the heart. However, the incidence of left ventricular remodeling (LVR) in PPGL is unclear.

Hypertension (HT) is the most common symptom of PPGL and can be recognized easily [3]. A previous study found that patients with PPGL had an increased left ventricular mass index (LVMI) and a higher frequency of left ventricular hypertrophy (LVH) independent of blood pressure level [4]. Another study showed that cardiac changes regressed significantly after adrenalectomy in patients with PPGL when compared with those with essential HT [5]. However, it is presently unknown whether a history of HT worsens the changes in cardiac structure and function that occur in PPGL.

In this study, we retrospectively compared patient demographics, reason for hospitalization, clinical characteristics, and cardiac structure and function in a population of PPGL patients according to whether or not they had a history of HT and examined the association between HT and left ventricular structure and function in these patients.

## Materials and methods

### Study population

The study participants were 598 patients who were treated for PPGL at Peking University First Hospital (Beijing, China) between January 2001 and April 2022. The following inclusion criteria were applied: laparotomy or laparoscopic surgery performed; a pathological diagnosis of PPGL; and complete perioperative data available in the electronic medical records. Information on demographics, medical history, reasons for hospitalization, results of biochemical examinations, findings on preoperative echocardiography, and tumor characteristics (Tumor size was calculated as the longest length of the tumor) was collected. The cause of hospitalization for health examination or health check-ups was regarded as incidental finding for PPGL. Patients were grouped according to history of HT, which was defined as a self-reported diagnosis of HT (office SBP values ≥ 140 mmHg and/or DBP values ≥ 90 mmHg) or use of antihypertensive medication, or hospitalization for BP elevation (office SBP values ≥ 140 mmHg and/or DBP values ≥ 90 mmHg).

The study was approved by the Peking University First Hospital Ethics Committee and conducted in accordance with the Declaration of Helsinki. The requirement for written informed consent was waived in view of the retrospective nature of the research by Peking University First Hospital Ethics Committee.

### Echocardiography exam

Transthoracic echocardiograms obtained before surgery were reviewed. All images and measurements were acquired from standard views according to the guidelines of the American Society of Echocardiography [6]. Relative wall thickness (RWT) was calculated using the following equation: (2 × left ventricular posterior wall thickness)/left ventricular end-diastolic diameter. Left ventricular mass was calculated using the American Society of Echocardiography cube formula. Left ventricular mass index (LVMI) was calculated by dividing left ventricular mass by body surface area. LVH was defined as an LVMI > 95 g/m^2^ in women and > 115 g/m^2^ in men [6]. LVR, including eccentric hypertrophy, concentric remodeling, and concentric hypertrophy, was defined as RWT > 0.42 or LVH in accordance with the guidelines of the American Society of Echocardiography [6].

### Statistical analysis

Continuous data are presented as the mean ± standard deviation for normal distributed variables and as the median [interquartile range] for non-normally distributed variables. Categorical variables are summarized as a number (percentage). Differences between two groups were compared using the Student’s t-test or chi-squared test. Demographics and clinical variables that were considered clinically relevant or showed a univariate relationship with LVMI/LVH/LVR were entered into a multivariate regression model. The statistical analysis was performed using the R (The R Foundation for Statistical Computing; http://www.r-project.org; version 4.2.0) and Empower (X&Y solutions, Inc., Boston, MA, USA; www.empowerstates.com) statistical software packages. All *P*-values were two-sided and considered statistically significant at < 0.05.

## Results

### Clinical characteristics and echocardiography findings of all PPGL patients

As shown in Table 1, the average age was 47.07 ± 15.07 years (range 9–82), and 277 (46.32%) of the patients were male; 423 (70.74%) had a history of HT on admission to hospital. One hundred and forty-one patients (23.58%) had paraganglioma. The most common reasons for hospitalization were findings on a health check-up (*n* = 201, 33.61%), HT (*n* = 241, 40.30%), the symptom triad of headache, palpitations, and sweating (*n* = 65, 10.87%), low back pain or abdominal pain (*n* = 53, 8.86%), headache or dizziness (*n* = 44, 7.36%) and cardiovascular symptoms (*n* = 43, 7.19%).

**Table 1:** Demographics and clinical characteristics of the study population

Variables	Total (*n* = 598)	Without HT history (*n* = 175)	With HT history (*n* = 423)	*P* value
Age, years	47.07 ± 15.07	44.95 ± 15.27	47.95 ± 14.92	0.027
Sex, male, n (%)	277 (46.32)	77 (44.00)	200 (47.28)	0.464
BMI, kg/m^2^	23.56 ± 3.40	23.16 ± 3.41	23.73 ± 3.39	0.063
Highest SBP before surgery, mmHg	165.51 ± 39.28	130.88 ± 17.98	179.84 ± 36.69	< 0.001
Highest DBP before surgery, mmHg	98.34 ± 24.11	78.59 ± 12.02	106.51 ± 23.11	< 0.001
Smoke, n (%)	81 (13.55)	19 (10.86)	62 (14.66)	0.217
Drink, n (%)	51 (8.53)	7 (4.00)	44 (10.40)	0.011
Stroke, n (%)	23 (3.85)	3 (1.71)	20 (4.74)	0.080
Diabetes, n (%)	108 (18.06)	16 (9.14)	92 (21.75)	< 0.001
Creatinine, µmol/L	76.19 ± 19.79	72.61 ± 16.11	77.65 ± 20.96	0.005
Cause of hospitalization, n (%)				
health examination	201 (33.61)	104 (59.43)	97 (22.93)	< 0.001
HT	241 (40.30)	0 (0.00)	241 (56.97)	< 0.001
triad symptom	65 (10.87)	8 (4.57)	57 (13.48)	0.001
waist or abdominal pain	53 (8.86)	29 (16.57)	24 (5.67)	< 0.001
headache or dizziness	44 (7.36)	7 (4.00)	37 (8.75)	0.043
cardiovascular symptoms	43 (7.19)	13 (7.43)	30 (7.09)	0.885
fever	8 (1.34)	5 (2.86)	3 (0.71)	0.038
tumor recurrence	11 (1.84)	2 (1.14)	9 (2.13)	0.415
urinary symptoms	10 (1.67)	7 (4.00)	3 (0.71)	0.004
Tumor				
tumor location, paraganglioma, n (%)	141 (23.58)	31 (17.71)	110 (26.00)	0.030
tumor size, cm	5.00 (3.80-7.00)	5.00 (3.50–6.50)	5.00 (4.00–7.00)	0.346

HT: hypertension, BMI: body mass index

Complete echocardiography data were available for 365 patients (61.04%), and the main findings are shown in Table 2. The average LVMI was 83.71 ± 24.99 g/m^2^ and the mean RWT was 0.43 ± 0.07. LVH was found in 60 patients (16.44%) and LVR (including eccentric hypertrophy, concentric remodeling, and concentric hypertrophy) in 182 (49.86%). Only eight patients (2.21%) had a decreased left ventricular ejection fraction.

**Table 2:** Echocardiographic findings in the study population

Variables	Total (*n* = 365)	Without HT history (*n* = 97)	With HT history (*n* = 268)	*P* value
LAD (cm)	3.23 ± 0.49	3.16 ± 0.50	3.26 ± 0.49	0.070
LVPW (cm)	0.96 ± 0.15	0.91 ± 0.12	0.97 ± 0.16	< 0.001
IVS (cm)	0.98 ± 0.17	0.92 ± 0.15	1.00 ± 0.17	< 0.001
LVEDD (cm)	4.50 ± 0.49	4.43 ± 0.40	4.53 ± 0.52	0.114
LVESD (cm)	2.77 ± 0.49	2.70 ± 0.37	2.80 ± 0.53	0.107
RWT	0.43 ± 0.07	0.41 ± 0.06	0.43 ± 0.08	0.012
LVFS (%)	0.40 ± 0.10	0.39 ± 0.05	0.40 ± 0.12	0.393
LVEF < 50% (%)	8 (2.21)	1 (1.03)	7 (2.64)	0.356
LVM (g)	150.55 ± 51.55	135.22 ± 35.97	156.10 ± 55.15	< 0.001
LVMI (g/m^2^)	83.71 ± 24.99	75.80 ± 17.26	86.58 ± 26.70	< 0.001
LVH, n (%)	60 (16.44)	8 (8.25)	52 (19.40)	0.011
LVR, n (%)	182 (49.86)	38 (39.18)	144 (53.73)	0.014
E-wave (cm/s)	80.16 ± 21.32	83.10 ± 25.14	79.08 ± 19.68	0.114
A-wave (cm/s)	80.43 ± 19.32	74.09 ± 17.20	82.74 ± 19.56	< 0.001
E/A	1.05 ± 0.37	1.16 ± 0.39	1.01 ± 0.36	< 0.001
E’ (cm/s)	7.56 ± 2.57	8.26 ± 2.65	7.27 ± 2.49	0.004
E/e’	11.28 ± 4.63	10.71 ± 5.96	11.51 ± 3.95	0.202
PASP (mmHg)	26.83 ± 7.85	26.09 ± 4.42	27.12 ± 8.83	0.336
TR (cm/s)	230.95 ± 38.40	231.21 ± 30.57	230.85 ± 41.19	0.944

HT: hypertension, IVS: interventricular septum, LAD: left atrial diameter, LVEDD: left ventricular end-diastolic dimension, LVEF: left ventricular ejection fraction, LVFS: left ventricular fractional shortening, LVESD: left ventricular end-systolic diameter, LVH: left ventricular hypertrophy, LVM: left ventricular mass, LVMI: left ventricular mass index, LVPW: left ventricular posterior wall, LVR: left ventricular remodeling, PASP: pulmonary artery systolic pressure, RWT: relative wall thickness, TR: tricuspid regurgitation

### Comparison of clinical characteristics and echocardiography findings according to HT history

The patients were divided into two groups according to whether or not they had a history of HT. Patients without a history of HT tended to be younger (44.95 ± 15.27 years vs. 47.95 ± 14.92 years, *P* = 0.027). Findings on a health check-up (59.43% vs. 22.93%, *P* < 0.001), low back pain or abdominal pain (16.57% vs. 5.67%, *P* < 0.001), fever (2.86% vs. 0.71%, *P* = 0.038), and urinary symptoms (4.00% vs. 0.71%, *P* = 0.004) were more common reasons for hospital admission in patients without a history of HT. However, the prevalence of headache or dizziness (8.75% vs. 4.00%, *P* = 0.043) and of the symptom triad (13.48% vs. 4.57%, *P* = 0.001) was significantly higher in the group with a history of HT than in the group without. Significant between-group differences were also found in alcohol consumption (4.00% vs. 10.40%, *P* = 0.011), diabetes (9.14% vs. 21.75%, *P* < 0.001), creatinine level (72.61 ± 16.11 µmol/L vs. 77.65 ± 20.96 µmol/L, *P* = 0.005), and tumor location (17.71% vs. 26.00%, *P* = 0.030).

Analysis of echocardiographic parameters indicated that the group with a history of HT had a significantly larger interventricular septum (1.00 ± 0.17 cm vs. 0.92 ± 0.15 cm, *P* < 0.001), left ventricular posterior wall (0.97 ± 0.16 cm vs. 0.91 ± 0.12 cm, *P* < 0.001), RWT (0.43 ± 0.08 vs. 0.41 ± 0.06, *P* = 0.012), and LVMI (86.58 ± 26.70 vs. 75.80 ± 17.26, *P* < 0.001). Proportions of LVH and LVR were significantly higher in the group with a history of HT (19.40% vs. 8.25%, *P* = 0.011, and 53.73% vs. 39.18%, *P* = 0.014, respectively). The characteristics of the patients who had complete echocardiography data available are compared between the groups in Supplementary Table 1.

### Association between HT history and LVR

Table 3 shows the results of univariate and multivariate analyses of the relationship between history of HT and LVH/LVMI/LVR. After adjusting for age and gender, a history of HT was positively associated with LVH (odds ratio [OR] 3.03, 95% confidence interval [CI] 1.36–6.75; *P* = 0.007), which was in accordance with the result of univariate analysis (OR 2.68, 95% CI 1.22–5.87; *P* = 0.014). Univariate correlations of LVH/LVMI/LVR included demographic and clinical characteristics and are shown in Supplementary Table 2. After additional adjustment for BMI, alcohol, smoking, diabetes, stroke, creatinine, tumor location and tumor size on the basis of Model 1, the association remained the same (OR 2.71, 95% CI, 1.18–6.19; *P* = 0.018). The results for LVR in univariate analysis (OR 1.80, 95% CI 1.12–2.89; *P* = 0.015) and multivariate analysis (OR 1.83, 95% CI 1.11–3.03; *P* = 0.018) were in accordance with the findings reported above.

**Table 3:** Association between history of hypertension and LVH/LVMI/LVR

Variables	Non-adjusted		Model 1		Model 2	
	OR (95% CI)/β (95% CI)	*P*-value	OR (95% CI)/β (95% CI)	*P*-value	OR (95% CI) / β (95% CI)	*P*-value
LVMI	10.78 (5.08, 16.49)	< 0.001	10.17 (4.42, 15.92)	< 0.001	8.96 (3.11, 14.80)	0.003
LVH	2.68 (1.22, 5.87)	0.014	3.03 (1.36, 6.75)	0.007	2.71 (1.18, 6.19)	0.018
LVR*	1.80 (1.12, 2.89)	0.015	1.74 (1.08, 2.81)	0.024	1.83 (1.11, 3.03)	0.018

Model 1 has been adjusted for age and sex. Model 2 includes the factors from model 1 with further adjustment for body mass index, alcohol consumption, smoking status, diabetes, stroke, creatinine level, tumor location, and tumor size. *LVR includes eccentric hypertrophy, concentric remodeling, and concentric hypertrophy

CI: confidence interval, LVH: left ventricular hypertrophy, LVMI: left ventricular mass index, LVR: left ventricular remodeling, OR: odds ratio

## Discussion

In this study, we investigated the clinical characteristics of 598 PPGL patients.and found that PPGL was more likely be found incidentally during a health check-up in patients without a history of HT. Although the symptom triad of headache, palpitation, and sweating were considered classical manifestations of PPGL [7], it was more commonly found in the group with a history of HT and only a minority of our patients had them simultaneously. Among 365 patients with complete echocardiography data, patients with a history of HT seemed to be more likely to have LVH.

Incidentalomas were becoming increasingly common with the increasing use of abdominal ultrasonography and computed tomography in health check-ups. According to a Denmark study the rate was 25.3% for incident PPGL [8], while 29.4% in a Germany cohort of 201 patients [9]. In our study, 33.61% of PPGL were detected during a routine health check-up. However, considering that our study was conducted in a tertiary referral center, referral bias cannot be excluded, and the proportion of asymptomatic patients and true number of PPGL diagnosed during routine health check-ups might have been underestimated. Furthermore, extra-adrenal tumors may need more screening, especially in asymptomatic patients with a history of HT, to avoid missed diagnoses.

The chronic pressure overload caused by HT can induce cardiac damage. One of the characteristic changes was hypertrophic growth of cardiomyocytes and progression of LVH/LVR [10]. In total, 423 (70.74%) of the 598 patients in our study had a history of HT at the time of admission. Some studies examined LVMI in patients with PPGL, comparing it to those with essential HT, but there was still disagreement. According to a Bulgarian research [11], LVH appeared more common in PPGL than essential HT (75% vs. 17%, *P* < 0.001). Nevertheless, the difference was not significant in other studies [5, 12]. In our large-scale hospital-based cohort study, we divided our patients into two groups according to whether or not they had a history of HT and after adjustment for confounders and found that LVH/LVR was more likely when there was a history of HT.

Catecholamines may play an important role in LVR in PPGL patients with HT history. A study in Poland found a positive correlation of LVMI with plasma neuropeptide Y immunoreactivity but not with plasma or urinary noradrenaline or adrenaline levels in patients with pheochromocytoma [13]. Another study retrospectively identified a relationship between secretion mode of noradrenergic phenotype and a reverse dipping status and LVH in PPGL patients [14]. A Chinese study also suggested that the 24-h urinary norepinephrine value was positively associated with the LVMI [15].

Unfortunately, our single-center study had very limited data for metanephrine and normetanephrine because the test was not routinely conducted until recent years. To account for catecholamines as a potential confounder, we conducted additional analysis in Supplementary Table 3. Although catecholamines data were incomplete, our main results remained unchanged. Moreover, it has been reported that the plasma level of adrenomedullin, a peptide produced by pheochromocytoma, is positively correlated with LVMI [16], which might also help to explain the high prevalence of LVH in PPGL patients. Although adrenomedullin is not considered to be a predictor of hemodynamic instability in adrenalectomy [17], its level in plasma might provide additional evidence of LVMI. PPGL patients with severe complications, such as catecholamine cardiomyopathy and Takotsubo-like cardiomyopathy, are also likely to have LVH [18]. Follow-up studies indicate that adrenalectomy can slow the progression of LVR [4, 5, 11, 19], which suggests that PPGL patients with LVH are likely to benefit from surgery. The current study was lack of sufficient metanephrine and normetanephrine data, which we expect to fill this regret in the future study. We would also investigate the change for LVR/LVH by follow up with an echocardiography after surgery.

This study has several limitations. First, the study was performed at a single center so is inevitably prone to bias. Second, the study had a retrospective design. Therefore, it relied on electronic medical records, in which data on echocardiography findings and plasma or urinary catecholamine levels were incomplete and there were no 24-hour blood pressure monitoring data. Third, it was not possible to obtain data on genetic phenotypes. Fourthly, the test for metanephrine and normetanephrine was not routinely done until recent years, as such data analysis is using a smaller sample number and may be prone to error. Finally, follow-up has yet to be completed in the study population, which means that not all prognostic and outcomes data are available.

## Conclusions

The findings of this study indicate that HT is common in PPGL patients. A history of HT was associated with LVMI/LVH/LVR in PPGL patients with complete echocardiography data. Patients with PPGL may have more severe cardiac damage if they have a history of HT and should be monitored very carefully in clinical practice.

### Electronic supplementary material

Below is the link to the electronic supplementary material.

Supplementary Material 1

## Data Availability

The data that support the findings of this study are available from the corresponding author upon reasonable request after the request is submitted and formally reviewed and approved by the ethics committee of Peking University First Hospital.
