# Supplementary material for: Hypertension and cardiac damage in pheochromocytoma and paraganglioma patients: a large-scale single-center cohort study
Source: BMC Cardiovasc Disord. 2024 Jun 26;24:325. doi: 10.1186/s12872-024-03936-6 (PMC11200840; doi:10.1186/s12872-024-03936-6)
Supplement: Supplementary file 1 — Supplementary Material 1 [file 12872_2024_3936_MOESM1_ESM.docx]

**Supplement**

Title : Hypertension and cardiac damage in pheochromocytoma and paraganglioma patients: a large-scale single-center cohort study

Yang Yu^1, 2†^, Chuyun Chen^1†^, Lei Meng^1, 2^, Wencong Han^3^, Yan Zhang^1, 4^, Zheng Zhang^3, 5, 6*^, Ying Yang^1, 2*^

* Corespondence: [doczhz@aliyun.com](mailto:doczhz@aliyun.com), [yangying1527@163.com](mailto:yangying1527@163.com)

^†^These authors contributed equally to this work and share first authorship

^1^Department of Cardiology, Peking University First Hospital, Beijing, China

^2^Echocardiography Core Lab, Institute of Cardiovascular Disease, Peking University First Hospital, Beijing, China

^3^Department of Urology, Peking University First Hospital, Beijing, China

^4^Institute of Cardiovascular Disease, Peking University First Hospital, Beijing, China

^5^Institute of Urology, Peking University, Beijing, China

^6^National Urological Cancer Center, Peking University First Hospital, Beijing, China

- **Supplementary Table 1.** Demographic and clinical characteristics of patients with complete echocardiography data
- **Supplementary Table 2. Results of univariate analysis.**
- **Supplementary Table 3. Results of multivariate analysis.**

**Supplementary Table 1 Demographic and clinical characteristics of patients with complete echocardiography data**

| **Variables** | **Total (n=365)** | **Without HT history (n=97)** | **With HT history (n=268)** | **P value** |
| --- | --- | --- | --- | --- |
| Age, years | 48.65 ±15.45 | 45.36 ±15.64 | 49.84 ±15.24 | 0.014 |
| Sex, male, n (%) | 169 (46.30) | 38 (39.18) | 131 (48.88) | 0.100 |
| BMI, kg/m^2^ | 23.53 ±3.29 | 22.72 ±2.95 | 23.83 ±3.36 | 0.004 |
| Smoke, n (%) | 62 (16.99) | 13 (13.40) | 49 (18.28) | 0.273 |
| Drink, n (%) | 42 (11.51) | 3 (3.09) | 39 (14.55) | 0.002 |
| Stroke, n (%) | 16 (4.40) | 3 (3.09) | 13 (4.87) | 0.465 |
| Diabetes, n (%) | 77 (21.10) | 11 (11.34) | 66 (24.63) | 0.006 |
| Creatinine, µmol/L | 76.62 ±22.00 | 70.48 ±14.73 | 78.83 ±23.72 | 0.001 |
| Cause of hospitalization, n (%) |  |  |  |  |
| health examination | 112 (30.68) | 53 (54.64) | 59 (22.01) | <0.001 |
| HT | 150 (41.10) | 0 (0.00) | 150 (55.97) | <0.001 |
| triad symptom | 38 (10.41) | 4 (4.12) | 34 (12.69) | 0.018 |
| waist or abdominal pain | 30 (8.22) | 15 (15.46) | 15 (5.60) | 0.002 |
| headache or dizziness | 36 (9.86) | 5 (5.15) | 31 (11.57) | 0.070 |
| cardiovascular symptoms | 39 (10.68) | 11 (11.34) | 28 (10.45) | 0.807 |
| fever | 5 (1.37) | 4 (4.12) | 1 (0.37) | 0.006 |
| tumor recurrence | 8 (2.19) | 2 (2.06) | 6 (2.24) | 0.919 |
| urinary symptoms | 6 (1.64) | 3 (3.09) | 3 (1.12) | 0.190 |
| elevation of plasma catecholamine* |  |  |  |  |
| epinephrine | 50/166 | 11/42 | 39/124 | 0.521 |
| norepinephrine | 135/168 | 31/41 | 104/127 | 0.379 |
| dopamine | 19/152 | 7/39 | 12/113 | 0.233 |
| MN | 43/122 | 13/41 | 30/81 | 0.561 |
| NMN | 90/123 | 30/41 | 60/82 | 1.000 |
| Tumor |  |  |  |  |
| paraganglioma, n (%) | 79 (21.64) | 15 (15.46) | 64 (23.88) | 0.085 |
| tumor size, cm | 5.00 (3.59-6.50) | 4.70 (3.50-6.00) | 5.00 (3.90-6.90) | 0.412 |

*Plasma adrenaline, noradrenaline, and dopamine were recorded in 166, 168, and 152 participants, respectively. Elevation of plasma epinephrine, norepinephrine and dopamine was deﬁned as beyond the upper limit of the normal range. Elevation of plasma MN and NMN was deﬁned as beyond the two times of the normal upper limit range. BMI, body mass index; HT, hypertension; MN, metanephrine; NMN, normetanephrine.

**Supplementary Table 2 Results of univariate analysis**

| **Variables** | **LVMI** | | **LVH** | | **LVR** | |
| --- | --- | --- | --- | --- | --- | --- |
|  | **β (95% CI)** | **P value** | **OR(95% CI)** | **P value** | **OR(95% CI)** | **P value** |
| Age | 0.04 (-0.13, 0.20) | 0.678 | 1.00 (0.98, 1.02) | 0.762 | 1.01 (1.00, 1.03) | 0.058 |
| Sex, Male | 6.72 (1.62, 11.82) | 0.010 | 0.40 (0.22, 0.73) | 0.003 | 0.95 (0.63, 1.43) | 0.790 |
| BMI | 1.05 (0.28, 1.83) | 0.008 | 1.01 (0.93, 1.10) | 0.849 | 1.01 (0.95, 1.07) | 0.823 |
| Smoke | 10.52 (3.77, 17.27) | 0.002 | 1.12 (0.54, 2.30) | 0.761 | 0.93 (0.54, 1.61) | 0.799 |
| Drink | 4.06 (-3.98, 12.09) | 0.323 | 0.66 (0.25, 1.75) | 0.402 | 0.73 (0.38, 1.39) | 0.336 |
| Stroke | 14.08 (1.61, 26.54) | 0.028 | 1.20 (0.33, 4.36) | 0.778 | 1.32 (0.48, 3.61) | 0.595 |
| Diabetes | 3.08 (-3.21, 9.36) | 0.338 | 1.17 (0.60, 2.26) | 0.642 | 1.11 (0.67, 1.84) | 0.680 |
| Creatinine | 0.14 (0.03, 0.26) | 0.017 | 1.00 (0.99, 1.02) | 0.387 | 1.00 (0.99, 1.01) | 0.543 |
| elevation of plasma catecholamine* |  |  |  |  |  |  |
| epinephrine | -2.66 (-11.76, 6.44) | 0.568 | 0.96 (0.42, 2.19) | 0.920 | 1.18 (0.61, 2.29) | 0.632 |
| norepinephrine | 0.59 (-9.91, 11.09) | 0.913 | 1.29 (0.49, 3.40) | 0.613 | 0.90 (0.42, 1.93) | 0.792 |
| dopamine | -6.37 (-19.78, 7.05) | 0.354 | 0.46 (0.10, 2.12) | 0.321 | 1.35 (0.52, 3.54) | 0.540 |
| Tumor |  |  |  |  |  |  |
| tumor location, paraganglioma | 8.53 (2.36, 14.70) | 0.007 | 2.08 (1.14, 3.83) | 0.018 | 1.54 (0.93, 2.54) | 0.094 |
| tumor size | 1.08 (0.14, 2.01) | 0.025 | 1.12 (1.02, 1.22) | 0.019 | 1.03 (0.96, 1.11) | 0.420 |

*Plasma adrenaline, noradrenaline, and dopamine were only recorded in 166, 168, and 152 participants, respectively. BMI, body mass index; CI, confidence interval; LVH, left ventricular hypertrophy; LVMI, left ventricular mass index; LVR, left ventricular remodeling; OR, odds ratio

**Supplementary Table 3 Results of multivariate analysis**

Considering that excessive secretion of noradrenaline might be associated with myocardial injury in PPGL ^[1-2]^, it was further adjusted in Model 3.

| **Variables** | **Non-adjusted** | | **Model 1** | | **Model 2** | | **Model 3** | |
| --- | --- | --- | --- | --- | --- | --- | --- | --- |
|  | **OR (95% CI) / β (95% CI)** | **P value** | **OR (95% CI) / β (95% CI)** | **P value** | **OR (95% CI) / β (95% CI)** | **P value** | **OR (95% CI) / β (95% CI)** | **P value** |
| **LVMI** | 10.78 (5.08, 16.49) | <0.001 | 10.17 (4.42, 15.92) | <0.001 | 8.96 (3.11, 14.80) | 0.003 | 10.99 (1.64, 20.34) | 0.023 |
| **LVH** | 2.68 (1.22, 5.87) | 0.014 | 3.03 (1.36, 6.75) | 0.007 | 2.71 (1.18, 6.19) | 0.018 | 4.01 (1.04, 15.42) | 0.043 |
| **LVR** | 1.80 (1.12, 2.89) | 0.015 | 1.74 (1.08, 2.81) | 0.024 | 1.83 (1.11, 3.03) | 0.018 | 2.34 (1.02, 5.35) | 0.045 |

Model 1 was adjusted for age and sex. Model 2 was adjusted for the factors in model 1 as well as body mass index, alcohol consumption, smoking status, diabetes, stroke, creatinine level, tumor location, and tumor size. Model 3 was adjusted for the factors in model 2 with addition of noradrenaline. *LVR group includes eccentric hypertrophy, concentric remodeling, and concentric hypertrophy. CI, confidence interval; LVH, left ventricular hypertrophy; LVMI, left ventricular mass index; LVR, left ventricular remodeling; OR, odds ratio

**REFERENCE**

1. Ding L, Zhu WL, Zeng ZP, Li HZ, Ji J, Fang LG, et al. Subclinical left ventricular systolic dysfunction detected by two-dimensional speckle tracking echocardiography in patients with pheochromocytoma and paraganglioma and preserved ejection fraction. *Echocardiography* (2018) 35(2):184-89. doi: 10.1111/echo.13752

2.Petrák O, Rosa J, Holaj R, Štrauch B, Krátká Z, Kvasnička J, et al. Blood Pressure Profile, Catecholamine Phenotype, and Target Organ Damage in Pheochromocytoma/Paraganglioma. J Clin Endocrinol Metab (2019) 104(11):5170-80. doi: 10.1210/jc.2018-02644
